# The Prevalence of Metabolic Syndrome and Its Association With Waist Circumference in Middle-Aged Individuals From Urban Mumbai

**DOI:** 10.7759/cureus.69669

**Published:** 2024-09-18

**Authors:** Ashish Goel, Paula Goel, Saurabh Goel

**Affiliations:** 1 Department of Cardiology, Fayth Clinic, Mumbai, IND; 2 Department of Pediatrics, Fayth Clinic, Mumbai, IND; 3 Department of Cardiology, Wockhardt Hospital, Mumbai Central, Mumbai, IND

**Keywords:** ncep atp iii criteria, waist circumference, elevated blood pressure, obesity and diabetes, metabolic disease, metabolic syndrome (mets)

## Abstract

Background

Metabolic syndrome (MetS) represents a critical public health challenge globally, characterized by a cluster of metabolic abnormalities that heighten the risk of cardiovascular diseases and type 2 diabetes. In India, the prevalence of MetS, particularly in urban areas, is rising rapidly. This study investigates the prevalence of MetS and its association with waist circumference in middle-aged individuals from urban Mumbai.

Methods

A cross-sectional study was conducted among 1,851 participants (814 men and 1,037 women, with a mean age of 56.8 years) in a public health camp in urban Mumbai. Data were collected on anthropometric measures, blood pressure, and blood markers, including fasting glucose and lipid profiles. MetS was diagnosed based on the National Cholesterol Education Program Adult Treatment Panel III (NCEP ATP III) criteria. This included the presence of three or more of the following five criteria: waist circumference of ≥102 cm for men and ≥88 cm for women, fasting triglycerides of ≥150 mg/dL, fasting high-density lipoprotein (HDL) cholesterol of <40 mg/dL for men and <50 mg/dL for women, blood pressure of ≥130/85 mm Hg, and fasting glucose of ≥100 mg/dL. Data were analyzed using SPSS Statistics version 23 (IBM SPSS Statistics, Armonk, NY). Statistical analyses were performed using the chi-square test, with statistical significance set at p<0.05.

Results

The overall prevalence of metabolic syndrome (MetS) in the cohort was 32.6% (605 out of 1,851 participants), with women exhibiting a significantly higher prevalence at 38% (394 out of 1,037 women) compared to men at 26% (211 out of 814 men) (p<0.001). High waist circumference (≥102 cm for men and ≥88 cm for women) was strongly correlated with MetS, as 73.8% of individuals (314 out of 425 participants) in the high waist circumference group met the criteria for MetS, compared to 20.4% of individuals (291 out of 1,426 participants) in the non-high waist circumference group (<102 cm for men and <88 cm for women) (p<0.001). Furthermore, elevated blood pressure, elevated fasting glucose, and elevated fasting triglycerides were significantly more common in the high waist circumference group, than in the non-high waist circumference group (p<0.001).

Conclusion

The study highlights the significant association between central obesity and MetS in an urban Indian population, with notably higher prevalence in women. Waist circumference is a critical determinant of MetS and should routinely be measured, with significant application especially in resource-limited settings for early detection and intervention.

## Introduction

Metabolic syndrome (MetS) has emerged as a critical public health issue worldwide. It is characterized by a constellation of metabolic abnormalities including central obesity, hypertension, dyslipidemia, and impaired glucose tolerance. The syndrome significantly elevates the risk of developing type 2 diabetes and cardiovascular diseases, thereby contributing to increased morbidity and mortality rates. In India, the prevalence of MetS has been rising steadily, particularly in urban areas. A recent study highlighted that approximately one-third of urban Indians are affected by MetS, with the condition impacting women more than men [1]. This disparity may be attributed to differences in lifestyle, dietary habits, and genetic predispositions prevalent in urban settings [1]. Urbanization and industrialization processes have significantly altered the traditional lifestyle and dietary patterns of the Indian population. The increased consumption of high-calorie processed foods, sedentary lifestyles, and reduced physical activity are major contributing factors to the growing prevalence of MetS in urban areas​ [1,2]. Studies also indicate that the prevalence of MetS varies widely across different regions of India, with urban regions showing higher rates compared to rural areas​ ​[1]. Given these findings, it is imperative to focus on early detection and intervention strategies tailored to urban populations. Public health initiatives prioritizing lifestyle modifications, including dietary changes and increased physical activity, are greatly indicated. Additionally, there is a need for gender-specific health education programs to address the unique metabolic health challenges faced by women in India. Understanding regional and demographic variations in the prevalence of MetS can help in formulating targeted public health policies aimed at reducing the overall burden of this syndrome. The presented cross-sectional study seeks to explore the prevalence of MetS and its association with waist circumference among individuals in urban Mumbai, thereby contributing to the growing body of evidence necessary for effective health interventions.

## Materials and methods

Study design

This cross-sectional study was conducted in a public health camp in urban Mumbai. The participants were assessed for height, weight, BMI, blood pressure, and blood markers, including fasting blood glucose and fasting lipids. One thousand eight hundred fifty-one individuals participated in this study. The cohort comprised 814 men and 1,037 women and a mean age of 56.8 years.

Data collection

Standard protocols were used for the collection of demographic information, BMI measurements, blood pressure readings, and blood sample collection. Height was measured using a stadiometer, weight using a digital scale, and blood pressure using calibrated sphygmomanometers. Blood samples were collected after an overnight fast (12 hours) and analyzed in a certified laboratory for glucose and lipid levels.

Data collection protocols

Demographic Information

Informed consent forms were distributed prior to data collection, outlining the study's purpose and procedures. Participation was restricted to individuals with signed consent forms. A confidential questionnaire collected demographic information such as age, gender, ethnicity, and socioeconomic status.

Height and Weight Measurements (BMI)

Trained healthcare professionals or research assistants conducted height and weight measurements using standardized equipment in private settings to ensure participant comfort and confidentiality. BMI was calculated using the standard formula, BMI=weight (kg)/square of height in meters, and individuals were categorized based on the Asian and South Asian BMI classification [3]. Measurements were recorded on individual data forms, and the participants were assigned unique identifiers to maintain anonymity.

Blood Pressure Readings

Blood pressure readings were obtained using calibrated sphygmomanometers with appropriate cuff size. Measurements were taken in a quiet and comfortable environment to minimize potential stressors. Three separate readings are obtained for the participants documenting high blood pressure, with a brief rest period between measurements to reduce the potential for inaccurate readings due to stress or discomfort. The average of the three readings was used to determine the participant's blood pressure status.

Waist Circumference

Waist circumference was measured with standard measuring tapes and recorded in centimeters.

Ethical considerations

This study adhered to the ethical guidelines outlined by institutional review boards and research standards for human subjects. Informed consent and assent were obtained, confidentiality was strictly maintained, and potential risks were minimized with appropriate support. Personal information was kept separate from research data, with identifiers replaced by unique codes. Strict data security measures were implemented, and personnel underwent training to uphold participant confidentiality. By implementing these data collection protocols, the study aimed to ensure research integrity, prioritize participant well-being, and generate reliable and ethically obtained data for analysis.

Diagnosis of metabolic syndrome (MetS)

MetS was diagnosed based on the National Cholesterol Education Program Adult Treatment Panel III (NCEP ATP III) criteria, which include waist circumference of ≥102 cm for men and ≥88 cm for women, fasting triglycerides of ≥150 mg/dL, fasting high-density lipoprotein (HDL) cholesterol of <40 mg/dL for men and <50 mg/dL for women, blood pressure of ≥130/85 mm Hg, and fasting glucose of ≥100 mg/dL [4]. Metabolic syndrome was defined as the presence of three or more of the above-listed five criteria.

Data analysis

Data analysis was analyzed using the SPSS software version 23 (IBM SPSS Statistics, Armonk, NY). The chi-square test was used for data analyses, and statistical significance was set at p<0.05.

## Results

The study cohort consisted of a total of 1,851 individuals, comprising 814 men (44% of the study population) with a mean age of 54 years and 1,037 women (56% of the study population) with a mean age of 59 years. The overall mean age of the cohort was 56.8 years.

As shown in Table 1, the overall prevalence of metabolic syndrome (MetS) in the study cohort was 32.6% (605 out of 1,851 participants). A significant gender difference was observed in the prevalence rates of MetS. Among the 1,037 women who participated in the study, 38% (394 participants) were found to have MetS, whereas 26% (211 participants) of the 814 men were diagnosed with MetS. This difference in prevalence between men and women was statistically significant (p<0.001). The higher prevalence in women suggests that gender may play a critical role in the risk factors associated with MetS.

**Table 1:** Prevalence of MetS *The difference between the prevalence of MetS in men and women was statistically significant MetS: metabolic syndrome

Group	Prevalence (%)	P-value
Overall	605/1,851 (32.6%)	
Men	211/814 (26%)	5.14×10^-8^ (<0.001)*
Women	394/1,037 (38%)	

As shown in Table 2, the high waist circumference group (≥102 cm for men and ≥88 cm for women) included 183 men and 242 women, while the non-high waist circumference group (<102 cm for men and <88 cm for women) included 631 men and 795 women. Within the high waist circumference group, 66.1% of men (121 participants) and 79.7% of women (193 participants) had MetS. The difference in prevalence rates between men and women in the high waist circumference group was statistically significant (p<0.001). In the non-high waist circumference group, 14.2% of men (90 participants) and 25.3% of women (201 participants) had MetS. The difference in prevalence rates between men and women in the non-high waist circumference group was statistically significant (p<0.001). These findings indicate a higher prevalence of MetS among women, in both the high and non-high waist circumference groups.

**Table 2:** Waist circumference and MetS *The difference between the prevalence of MetS in men and women in the high waist circumference group was statistically significant **The difference between the prevalence of MetS in men and women in the non-high waist circumference group was statistically significant ***The difference between the prevalence of MetS in the high waist circumference and non-high waist circumference groups was statistically significant MetS: metabolic syndrome

Waist circumference	Men	Women	Prevalence of MetS (%)	Prevalence of MetS in men (%)	Prevalence of MetS in women (%)	P-value	P-value
High (≥102 cm for men and ≥88 cm for women)	183/814 (22.5%)	242/1,037 (23.4%)	314/425 (73.8%)	121/183 (66.1%)	193/242 (79.7%)	0.0022 (<0.001)*	5.10×10^-94^ (<0.001)***
Non-high (<102 cm for men and <88 cm for women)	631/814 (77.5%)	795/1,037 (76.6%)	291/1,426 (20.4%)	90/631 (14.2%)	201/795 (25.3%)	4.14×10^-7 ^(<0.001)**	

Overall, individuals in the high waist circumference group had a much higher prevalence of MetS at 73.8% (314 of 425 participants), while individuals in the non-high waist circumference group had a lower prevalence of MetS at 20.4% (291 of 1,426 participants). The difference in prevalence rates between the high and non-high waist circumference groups was statistically significant (p<0.001).

As per Table 3, the most prevalent component of MetS was elevated blood pressure (≥130/85 mm Hg), with 60% prevalence in the high waist circumference group (255 of 425 participants) compared to 25% in the non-high waist circumference group (356 of 1,426 participants). The difference in prevalence rates of elevated blood pressure between the high waist circumference and non-high waist circumference groups was statistically significant (p<0.001).

**Table 3:** MetS components and waist circumference *The difference between the prevalence of elevated blood pressure (BP) in the high waist circumference and non-high waist circumference groups was statistically significant **The difference between the prevalence of elevated fasting glucose in the high waist circumference and non-high waist circumference groups was statistically significant ***The difference between the prevalence of elevated fasting triglycerides (TG) in the high waist circumference and non-high waist circumference groups was statistically significant MetS: metabolic syndrome

MetS component	High waist circumference prevalence (%)	Non-high waist circumference prevalence (%)	P-value
Elevated BP of ≥130/85 mm Hg	255/425 (60%)	356/1,426 (25%)	4.47×10^-41^ (<0.001)*
Elevated fasting glucose of ≥100 mg/dL	234/425 (55%)	257/1,426 (18%)	1.24×10^-51^ (<0.001)**
Elevated fasting TG of ≥150 mg/dL	212/425 (50%)	285/1,426 (20%)	6.17×10^-34^ (<0.001)***

Elevated fasting glucose was the second most prevalent component, observed in 55% of the high waist circumference group (234 of 425 participants), compared to 18% in the non-high waist circumference group (257 of 1,426 participants). This difference in the prevalence of fasting glucose between the high waist circumference and non-high waist circumference groups was statistically significant (p<0.001).

Elevated fasting triglycerides were the third most prevalent component, observed in 50% of the high waist circumference group (212 of 425 participants), versus 20% in the non-high waist circumference group (285 of 1,426 participants). The difference in the prevalence of elevated fasting triglycerides between the high waist circumference and non-high waist circumference groups was statistically significant (p<0.001).

**Figure 1:**
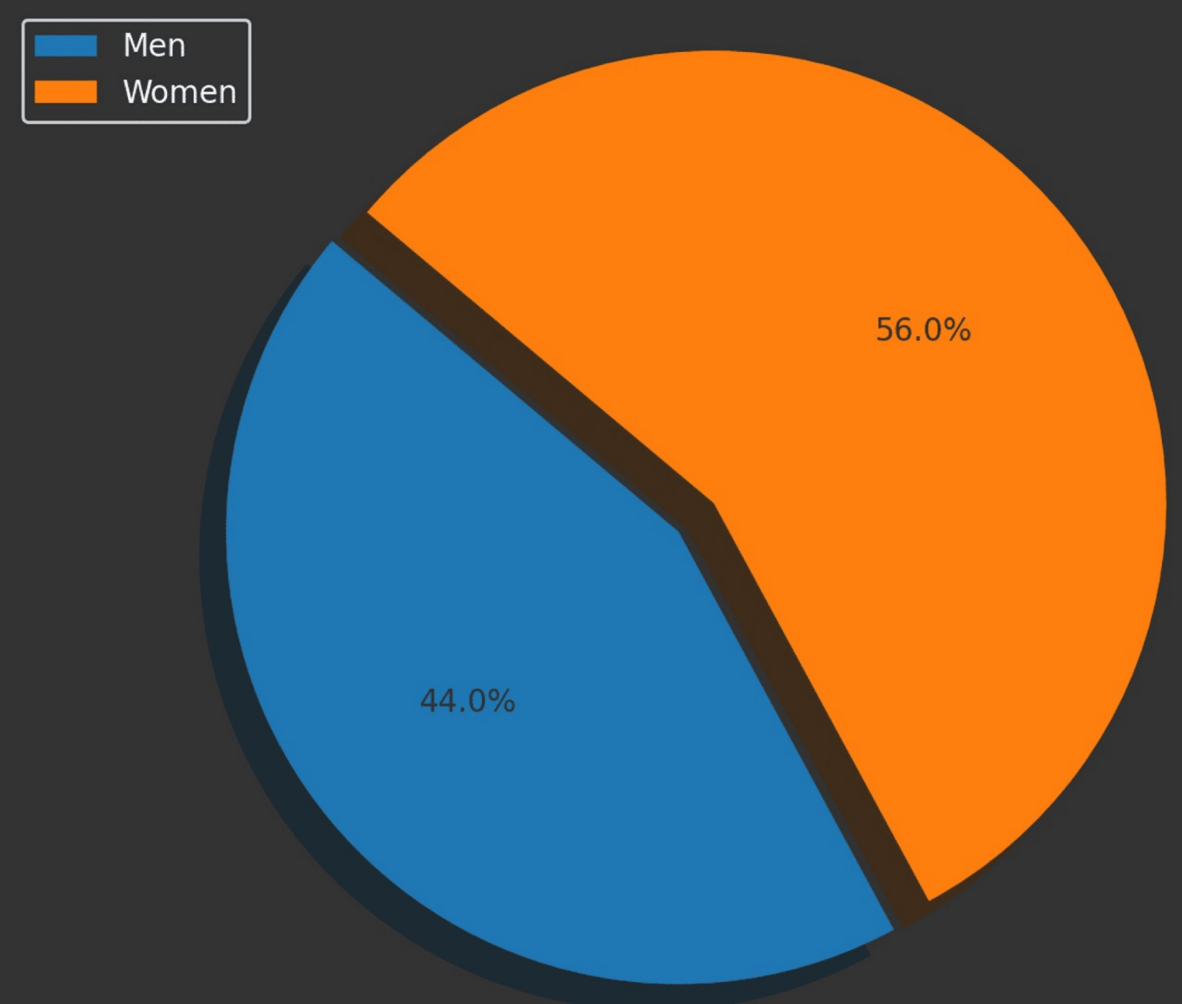
Gender distribution of MetS Men comprised 44% or 814 participants within the cohort (1,851 individuals). Women comprised 56% or 1,037 participants within the cohort (1,851 individuals) MetS: metabolic syndrome

**Figure 2:**
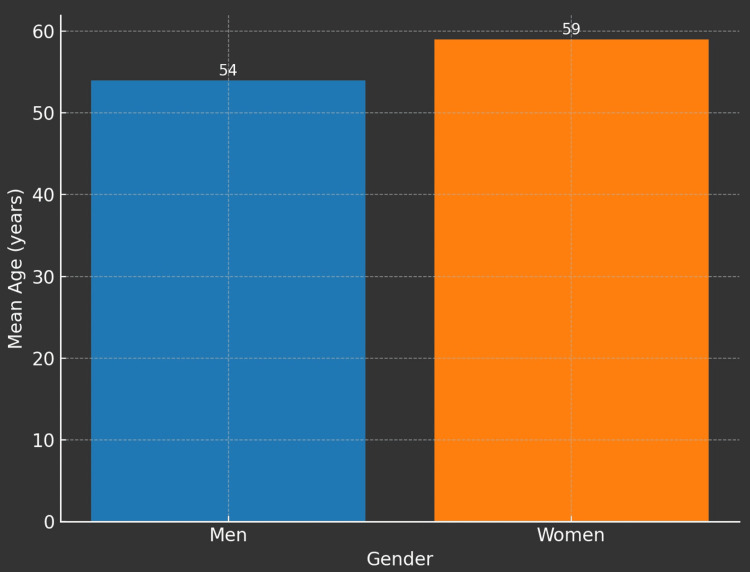
Mean age of the study participants

**Figure 3:**
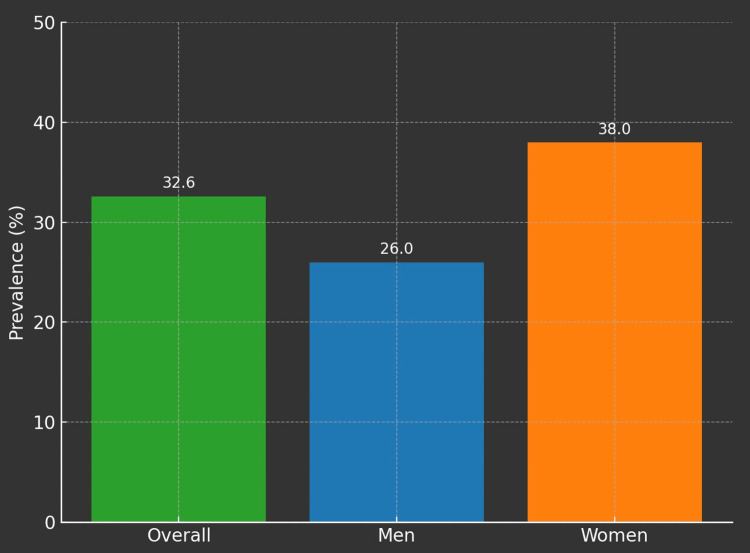
Prevalence of MetS among the study participants Overall prevalence of MetS, 32.6% or 605 (of 1,851) participants; prevalence of MetS in men, 26% or 211 (of 814) participants; and prevalence of MetS in women, 38% or 394 (of 1,037) participants MetS: metabolic syndrome

**Figure 4:**
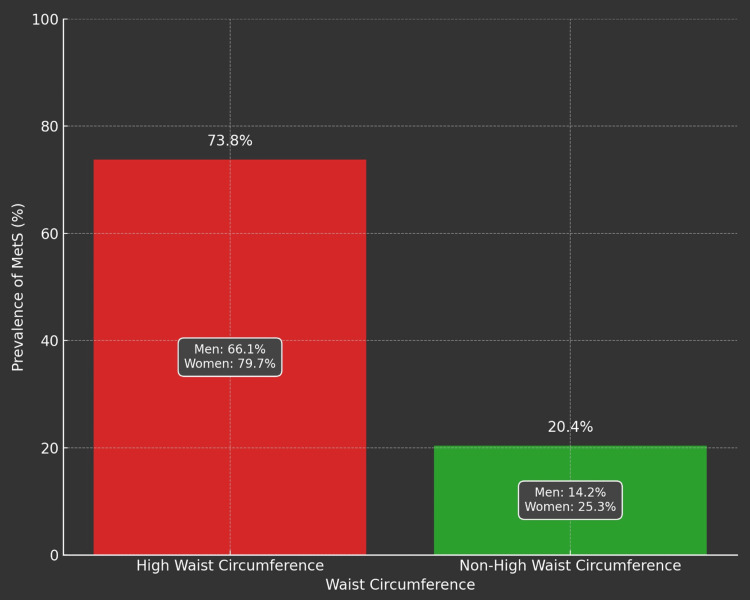
Prevalence of MetS based on waist circumference High waist circumference (≥102 cm for men and ≥88 cm for women): prevalence of MetS in the high waist circumference group, 73.8% or 314 (of 425) participants; prevalence of MetS among men within the high waist circumference group, 66.1% or 121 (of 183) participants; and prevalence of MetS among women within the high waist circumference group, 79.7% or 193 (of 242) participants Non-high waist circumference (<102 cm for men and <88 cm for women): prevalence of MetS in the non-high waist circumference group, 20.4% or 291 (of 1,426) participants; prevalence of MetS among men within the non-high waist circumference group, 14.2% or 90 (of 631) participants; and prevalence of MetS among women within the non-high waist circumference group, 25.3% or 201 (of 795) participants MetS: metabolic syndrome

**Figure 5:**
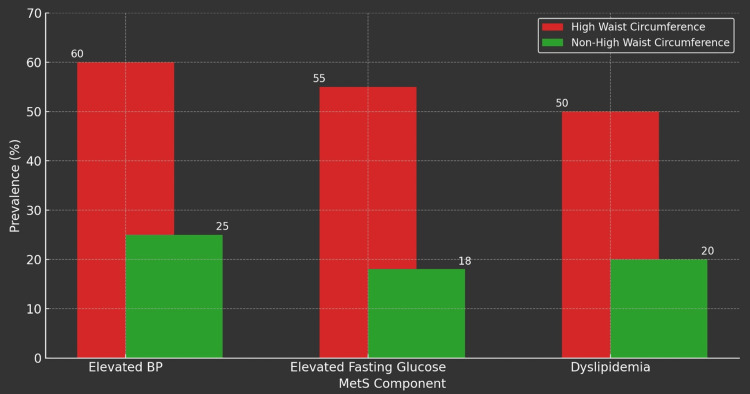
Prevalence of MetS among the study particpants based on waist circumference High waist circumference (≥102 cm for men and ≥88 cm for women) and non-high waist circumference (<102 cm for men and <88 cm for women) Blood pressure (BP) (≥130/85 mm Hg): prevalence of elevated BP within the high waist circumference group, 60% or 255 (of 425) participants; prevalence of elevated BP within the non-high waist circumference group, 25% or 356 (of 1,426) participants Elevated fasting glucose (≥100 mg/dL): prevalence of elevated fasting glucose within the high waist circumference group, 55% or 234 (of 425) participants; prevalence of elevated fasting glucose within the non-high waist circumference group, 18% or 257 (of 1,426) participants Dyslipidemia: elevated fasting triglycerides (TG) (≥150 mg/dL): prevalence of elevated fasting TG within the high waist circumference group, 50% or 212 (of 425) participants; prevalence of elevated fasting TG within the non-high waist circumference group, 20% or 285 (of 1,426) participants MetS: metabolic syndrome

Figures 1-5 visually represent the relevant trends and distributions observed in the study.

## Discussion

Our study highlights a significant association between central obesity and the prevalence of MetS in middle-aged individuals in India. Central or abdominal obesity is defined as a waist circumference greater than 80 cm in women and 94 cm in men [5]. It is a major determinant of metabolic syndrome (MetS), with a strong correlation observed between increased waist circumference and MetS components such as hypertension, hyperglycemia, and dyslipidemia [4,5]. The higher prevalence of MetS in women may be attributed to gender differences in central adiposity and hormonal influences [6,7].

Initially, MetS was primarily recognized in Western countries, but with increasing globalization and lifestyle changes, its prevalence has surged in developing nations, including India. This shift may be an unintended impact of urbanization and industrialization on health outcomes. Prevalence in India varies by region. Studies reported MetS prevalence rates of 19.76% in Kerala, 15.86% in Lakshadweep, 12.44% in Punjab, and 11.53% in Chandigarh, while the northeastern states such as Assam, Arunachal Pradesh, and Meghalaya have much lower rates, around 1%-2% [8]. This may indicate the influence of regional dietary habits, lifestyle, and genetic factors. Urbanization in certain areas of the northeastern states has been lower than elsewhere in the country, possibly contributing to the lower prevalence of MetS. Additionally, hilly regions within these parts encourage an active lifestyle among its population. As urbanization increases, there could be changes in lifestyle and dietary patterns that might impact these trends.

Physiologically, central obesity plays a crucial role in MetS by contributing to insulin resistance, which in turn leads to dyslipidemia, hypertension, and hyperglycemia. Central obesity is characterized by the accumulation of fat in the abdominal region, which is more metabolically active than subcutaneous fat [9,10]. This visceral fat is associated with a higher risk of developing metabolic disorders due to its proximity to the liver and other vital organs. The cytokines and adipokines released by visceral fat, including tumor necrosis factor-alpha (TNF-α), interleukin-6 (IL-6), and adiponectin, play significant roles in modulating insulin sensitivity, inflammation, and lipid metabolism [9-11].

Insulin resistance, a hallmark of MetS, is exacerbated by the increased secretion of free fatty acids from visceral fat. These free fatty acids interfere with insulin signaling pathways, leading to reduced glucose uptake by muscles and increased glucose production by the liver. The resulting hyperglycemia is a critical component of MetS and a precursor to type 2 diabetes [12]. Dyslipidemia in MetS is characterized by elevated levels of triglycerides and low-density lipoprotein (LDL) cholesterol, along with decreased levels of high-density lipoprotein (HDL) cholesterol. Visceral fat contributes to dyslipidemia by altering lipid metabolism and increasing the release of fatty acids into the bloodstream, further enhancing the risk of atherosclerosis and cardiovascular diseases [13,14].

Hypertension, another key feature of MetS, is influenced by the presence of central obesity through several mechanisms. Visceral fat increases the production of angiotensinogen, a precursor to angiotensin II, a potent vasoconstrictor, leading to increased blood pressure. Additionally, the pro-inflammatory state induced by visceral fat contributes to endothelial dysfunction, further exacerbating hypertension [15,16]. Apart from cardiovascular disease and type 2 diabetes, individuals with metabolic syndrome are at an increased risk for several other conditions, including polycystic ovary syndrome, fatty liver disease, cholesterol gallstones, asthma, sleep disorders, and certain types of cancer [4].

Given that central obesity significantly contributes to the overall metabolic burden, it serves as a practical target for intervention. Public health strategies focused on reducing central obesity through lifestyle modifications are effective in mitigating risk factors associated with MetS. Dietary changes, such as reducing refined carbohydrates and increasing fiber intake, combined with regular physical activity, can significantly reduce these risks [17]. Weight loss achieved through dietary changes, increased physical activity, and behavioral therapy has been shown to improve insulin sensitivity, lipid profiles, and blood pressure [18,19]. In addition to lifestyle modifications, pharmacological interventions also play a role. Agents such as metformin and glucagon-like peptide 1 (GLP-1) receptor agonists target weight reduction and improve metabolic parameters [20,21].

Studies have demonstrated that lifestyle interventions, including dietary modifications and physical activity, are effective in reducing the prevalence of MetS. For instance, a community-based intervention in South India showed a significant reduction in MetS prevalence through the promotion of healthier dietary practices and increased physical activity [22]. In addition to lifestyle interventions, pharmacological treatments may be necessary for individuals with severe MetS components. Medications targeting hypertension, dyslipidemia, and hyperglycemia can help manage the individual risk factors associated with MetS [23].

Given the high burden of MetS and its complications, including cardiovascular disease and type 2 diabetes, it is imperative to implement comprehensive public health strategies. These should include awareness campaigns, screening programs via public health camps, and interventions tailored to high-risk populations [24]. The urban middle-class population of Mumbai, characterized by sedentary lifestyles and dietary habits influenced by urbanization, presents a unique challenge. Public health campaigns should tailor their messages to address specific lifestyle factors and promote healthier living [25]. The significant gender disparity in MetS prevalence further suggests a need for gender-specific interventions [26,27]. Women, particularly in the post-menopausal age group, might benefit from targeted educational programs addressing hormonal changes and their impact on body fat distribution.

The treatment status of individuals with MetS also varies significantly. A systematic review and meta-analysis have shown that the pooled prevalence of MetS among adults in India is approximately 30%, with higher rates observed among urban populations, older adults, and women [22]. Another study highlighted that 8.85% of individuals with MetS and a previous diagnosis of diabetes or hypertension had not initiated treatment, while 17.58% were on partial treatment, and 73.56% were on full treatment [8]. These findings underline the importance of addressing MetS as a public health priority, especially in rapidly urbanizing regions, and the need for improved healthcare awareness and access and adherence to treatment protocols.

The economic and social implications of MetS are significant. The increased healthcare costs associated with managing MetS and its complications place a substantial burden on individuals and healthcare systems. For example, in relatively wealthy European countries such as Germany, Spain, and Italy, the costs of managing hypertension with MetS are projected to rise substantially, highlighting the growing economic strain on healthcare systems as the prevalence of MetS increases [28]. These costs are not limited to direct medical expenses but also include costs related to the treatment and management of associated diseases such as type 2 diabetes and cardiovascular conditions. In contrast, in resource-limited regions such as India, with population sizes that are substantially larger, these trends are only augmented.

A comparative analysis with other populations provides a global perspective on MetS. For instance, the prevalence of MetS varies significantly across different regions. In the Asia-Pacific region, the prevalence ranges widely, with the lowest reported in the Philippines at 11.9% and the highest in urban Pakistan at 49.0% [29]. Studies show higher prevalence rates in urban areas compared to rural ones, likely due to differences in lifestyle and dietary patterns. In China, recent national surveys reported a prevalence of 21.3%, with urban areas showing higher rates compared to rural regions. Similarly, in South Korea, the prevalence was 31.3%, and studies indicate an increasing trend over time [29]. In Middle Eastern countries, the prevalence of MetS is also high, with significant variations based on the criteria used for diagnosis. For example, in Iran, the prevalence ranges from 34.7% to 41.6%, depending on the diagnostic criteria, while in Tunisia, it ranges from 24.3% to 45.5% [30]. These variations are similar to our study's findings and highlight the need for more public health camps and studies in India to better understand regional variations, draw comparisons, and refine management strategies.

Limitations

The cross-sectional design of our study limits the ability to infer causality between body fat distribution and metabolic syndrome (MetS), as it captures data at a single point in time rather than over a period. Longitudinal studies would be required to establish causal relationships. Additionally, the participants were recruited from a public health camp, which may not be representative of the broader population of urban Mumbai. Those attending health camps might be more health-conscious or have preexisting health conditions. Furthermore, some data, such as dietary habits and physical activity levels, were self-reported by the participants. This self-reporting may introduce recall bias. Our study did not account for unmeasured variables, such as genetic predisposition, stress levels, and detailed dietary intake, that could influence the prevalence of MetS and its components. Conducting the study in a single urban center also limits the ability to generalize findings to other urban areas in India, which may have different lifestyle patterns and health profiles.

## Conclusions

The study found that the overall prevalence of MetS was 32.6% (605 of 1,851 participants), with a 38% prevalence in women (394 participants) compared to a 26% prevalence in men (211 participants). This gender disparity may be due to differences in body fat distribution and hormonal changes, particularly in post-menopausal women. MetS prevalence was significantly higher among those with high waist circumference (73.8% or 314 of 425 individuals) versus those without (20.4% or 291 of 1,426 individuals), highlighting central obesity's crucial role in MetS development. Individuals with high waist circumference were more likely to have elevated blood pressure, fasting glucose, and triglycerides, highlighting the strong link between central obesity and metabolic complications. The higher prevalence of MetS in women with high waist circumference supports the need for gender-specific management guidelines, especially in post-menopausal age group. Given the significant association between waist circumference and MetS, this study supports the use of waist circumference as an effective, low-cost screening tool, particularly in resource-limited areas. Further studies should investigate the proportion of individuals with undiagnosed MetS, those aware but untreated, and those receiving treatment. These groups could be analyzed across different regions to assess variations in MetS prevalence, awareness, and management between urban and rural populations of India. The routine measurement of waist circumference, especially in resource-constrained settings, could significantly bolster public health efforts to reduce the burden of MetS and its complications.
